# An Extensive Examination of the Warning Signs, Symptoms, Diagnosis, Available Therapies, and Prognosis for Lumpy Skin Disease

**DOI:** 10.3390/v15030604

**Published:** 2023-02-22

**Authors:** Bharti Datten, Anis Ahmad Chaudhary, Shalini Sharma, Lokender Singh, Krishna Dutta Rawat, Mohammad Saquib Ashraf, Lina M. Alneghery, Malak O. Aladwani, Hassan Ahmad Rudayni, Deen Dayal, Sanjay Kumar, Kundan Kumar Chaubey

**Affiliations:** 1 Department of Veterinary Physiology and Biochemistry, Lala Lajpat Rai University of Veterinary and Animal Sciences, Hisar 125004, Haryana, India; 2 Department of Biology, College of Science, Imam Mohammad Ibn Saud Islamic University (IMSIU), Riyadh 11623, Saudi Arabia; 3 Department of Bio and Nanotechnology, Guru Jambheshwar University of Science and Technology, Hisar 125001, Haryana, India; 4 Department of Medical Laboratory Sciences, College of Applied Medical Sciences, Riyadh ELM University, Riyadh 12734, Saudi Arabia; 5 Department of Biotechnology, GLA University, Mathura 281406, Uttar Pradesh, India; 6 Department of Life Science, Sharda School of Basic Sciences and Research, Sharda University, Knowledge Park-III, Greater Noida 201310, Uttar Pradesh, India; 7 Division of Research and Innovation, School of Applied and Life Sciences, Uttaranchal University, Dehradun 248007, Uttarakhand, India

**Keywords:** lumpy skin virus, cattle, symptoms, diagnosis, control, eradication, therapies, prognosis

## Abstract

The lumpy skin disease virus (LSDV) infects cattle and buffalo and causes lumpy skin disease (LSD). It affects the lymph nodes of the sick animals, causing them to enlarge and appear as lumps (cutaneous nodules) that are 2–5 cm in diameter on their heads, necks, limbs, udders, genitalia, and perinea. A high temperature, a sharp drop in milk supply, discharge from the eyes and nose, salivation, a loss of appetite, depression, damaged hides, and emaciation are further warning signs and symptoms. As per the Food and Agriculture Organization (FAO), the incubation period, or the time between an infection and symptoms, is approximately 28 days. Infected animals can transfer the virus by direct contact with the vectors, direct virus secretion from mouth or nose, shared feeding and watering troughs, and even artificial insemination. The World Organization for Animal Health (WOAH) and the FAO both warn that the spread of illnesses could lead to serious economic losses. This illness reduces cow’s milk production because oral ulcers make the animal weak and lead them to lose their appetite. There are many diagnostics available for LSDV. However, very few tests yield accurate findings. The best methods for preventing and controlling the lumpy skin condition include vaccination and movement restrictions. As a specific cure is not available, the only available treatment for this illness is supportive care for cattle. Recently, India has developed a homologous, live-attenuated vaccine, Lumpi-ProVacInd, which is specifically intended to protect animals against the LSD virus. This study’s primary goal is to accumulate data on symptoms, the most accurate method of diagnosis, treatments, and controls to stop infections from spreading as well as to explore future possibilities for the management of LSDV.

## 1. Introduction

Lumpy skin disease (LSD) has become a territorial infectious cattle disease that causes widespread destruction within the livestock industry, and it is registered as a notifiable disease by the World Organization for Animal Health (WOAH), formerly known as Office International des Epizooties (OIE). This is a serious skin disease that forms lumps of various sizes but mostly between 1 and 3 cm in diameter and 1 to 2 cm deep. The distribution of the lumps on the skin is random, ranging from a few lumps to hundreds [1].

It was first reported in Zambia in 1929 and spread quickly in the cattle community throughout African countries. LSD was retained in sub-Saharan Africa until 1984 [2]. The very first identified trans-continental transmission of LSD occurred from Africa to Middle East Asian countries in 1989, when the disease was confirmed in Israel [3]. According to the OIE, LSD was recorded in Kuwait in 1991, Lebanon in 1993, Yemen in 1995, United Arab Emirates in 2000, Israel in 2006–2007, and Oman in 2010. The disease was identified in Turkey in 2013. It was documented in the Balkans, South-East Europe, and the Caucasus by 2015–16. LSD was recorded for perhaps the first time in India in November 2019 [4].

**Figure 1:**
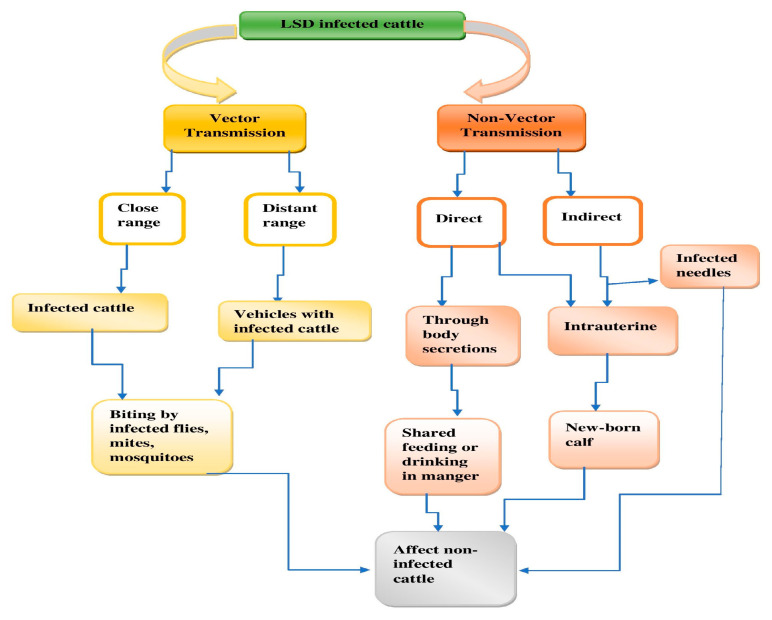
Transmission of LSDV.

The transmission pathways may vary among different genera within the Poxviridae family [5]. It is to be believed that LSD is usually transmitted via arthropod vectors. Intravenous inoculation of the LSD virus causes prominent clinical effects in experimentally susceptible hosts compared to intradermal infections, and therefore vectors such as mosquitoes that feed directly from blood vessels are more likely to be vector candidates [6]. The long-distance diffusion of LSDV tends to occur via the migration of infected animals. However, distinctive seasonal variations suggest that arthropod-borne transmission is still most probable to provoke the rapid and violent short-distance distribution of this disease (Figure 1). A hot and humid climate was correlated with increased proliferation of LSD, and Gari et al. (2010) suggested that all the PCse factors are associated with a higher vector population. Community feeding and watering sites were also linked with the prevalence of LSD. They also stated that the arrival of new member into a herd had quite a strong correlation with an increased risk of infection in the herd [7].

**Figure 2:**
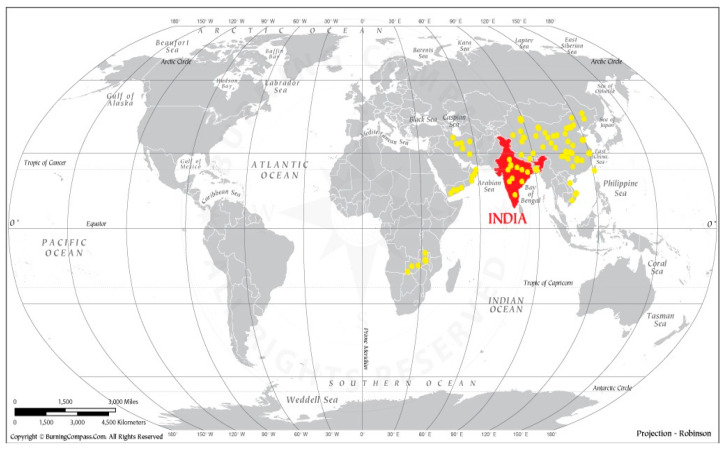
World political map indicating out breaks of LSDV in different countries.

**Figure 3:**
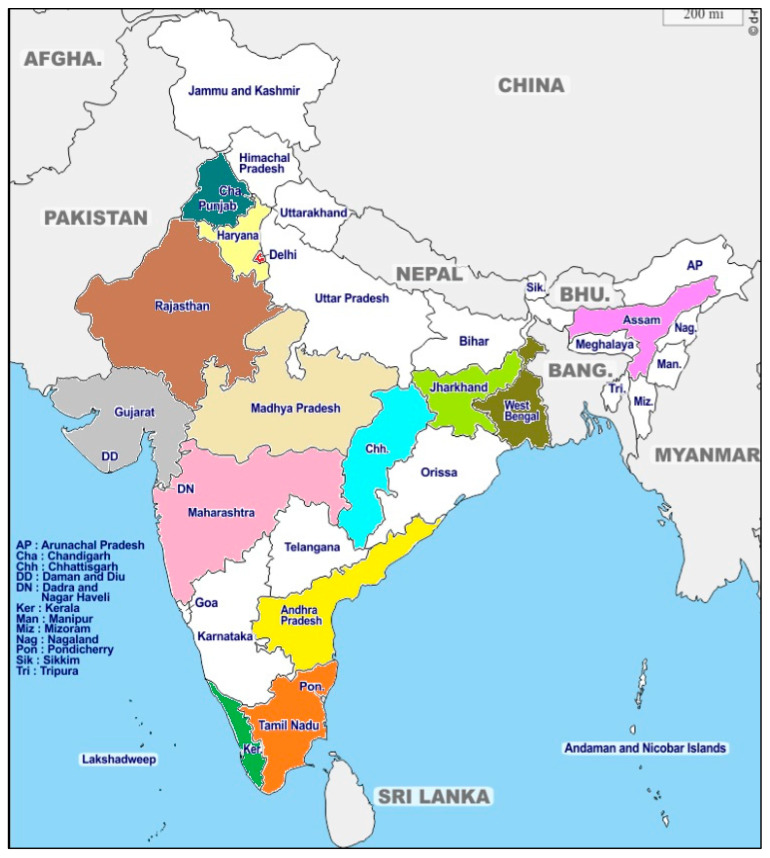
Indian political map indicating out breaks of LSDV in different states.

Initially, lumpy skin disease was limited to Africa for many decades. Later, it spread to several countries, affecting food security for cattle and thus increasing the severity of hunger affecting the region [8,9]. For the first time, LSD was detected in India in 2019 [4]. This outbreak has now expanded to many other states of India, viz. Gujarat, Rajasthan, Haryana, Delhi, Tamilnadu, Telangana, Orissa, Kerala, West Bengal, Maharashtra, Assam, Madhya Pradesh, Jharkhand, Andhra Pradesh, and Chhattisgarh, which has led to huge economic losses in the cattle industry. Therefore, the rapid and unusual expansion of this virus in India and many other countries drew attention to the scientific community for its proper management to protect cattle from this deadly pathogen (Figure 2; Table 1). The LSDV caused various outbreaks in Indian states, colored states indicating out breaks of LSDV in Indian political map (Figure 3).

## 2. Clinical Signs and Symptoms

Lumpy skin disease (LSD) is a trans-boundary viral infectious illness of cattle. The illness can affect cattle of any breed and age. However, depending on the virulence of the strains and the sensitivity of the cow breed, the severity of the clinical indications of LSD ranges from asymptomatic to lethal. Several clinical symptoms, including skin nodules, swollen lymph nodes, fever, nasal discharge, lacrimation, and mucous membrane and internal organ edema, might appear in infected animals [17,18].

Viremia and fever are the first symptoms of the generalized type, which are there after localized in the skin and produce inflammatory nodules [19]. The primary evidence of inflammation is lacrimation and fever, followed by skin lesions of varying proportions affecting the entire animal. In seriously affected cattle, ulcerative lesions develop in the mucosal surfaces of the eye and in the oral/nasal cavities, resulting in prolonged mastication, lacrimation, and nasal discharge [20]. Pox lesions can also be found in the larynx, lungs, pharynx, trachea, and the entire gut wall [20]. The incubation period for natural outbreaks was predicted to be 3 to 12 days [21].

Regardless of the method of infection, the interval between inoculation and the initial detection of widespread clinical indications in experimentally infected cattle spans from 7 to 14 days [2,22,23,24], and in naturally occurring instances it is between 2 and 5 weeks [14,15]. The temperature of the infected animals rises to 40–41.5 °C, which may last for six to seventy-two hours or more and, in exceptional cases, for up to ten days [2]. Additional symptoms of infection in animals include lacrimation, increased nasal and pharyngeal secretions, anorexia, dysgalactia, generalized sadness, and a lack of desire to move [17]. The severity of the early clinical manifestations of LSD varies, depending on the herd management method, and is unrelated to the sex or age of the animal. The epidermis of the animal develops several solid, bounded nodules [23]. Within 1–2 days, these nodules unexpectedly emerge. The nodules that have formed may be localized to a few lesions or may be extensive. The preferred locations include the head, neck, perineum, genitalia, udders, and limbs [24].

Clinical symptoms also include nodules on various regions of the body, including the udders, testicles, tail, back, neck, perineum, and limbs, that range in size from a few millimeters to 2–5 centimeters in diameter. Salivation, lameness, severe anorexia, stopping milk production, and mortality are other clinical indicators [2]. Brisket, edema of the limbs, and the expansion of the superficial lymph nodes are all very noticeable. Common eye conditions include opacity of the cornea, keratitis, and conjunctivitis. Nodules are found during autopsy on the tongue’s dorsum. Nodules are most often found on the head, neck, perineum, genitalia, legs, and udders; they affect the epidermis, dermis, and cutaneous tissues and, on rare occasions, an underlying muscle [25]. Anodular lesion shows as a spherical, hard, intradermal, and confined region of erected hair with a diameter of up to 1–7 cm [2]. In extreme cases, the mucous membranes of the mouth, trachea, larynx, and esophagus may develop ulcerative sores. The necrotic cores, often known as “sit-fasts”, detach from the surrounding skin.

The mortality, morbidity, and clinical manifestations of LSD include (a) contagious illness with widespread skin nodules; (b) skin nodules with the characteristic inverted conical necrosis (sit-fasts) and enlarged lymph nodes draining afflicted areas; (c) low mortality, emaciation, and ongoing fever; (d) pox lesions on the mucous membranes of the mouth, throat, tongue, epiglottis, gastrointestinal tract, nasal cavity, trachea, and lungs; (e) areas of localized lobular atelectasis and edema in the lungs; (f) pleuritis with mediastinal lymph node hypertrophy in severe cases; (g) fibrin in the synovial fluid in synovitis and tendosynovitis; (h) the testicles and bladder may have shingles lesions; and (i) early-stage skin lesions, which should be biopsied and kept in 10% buffered formalin for histopathology. LSD can be accompanied by afrequent bacterial infection and a typically deadly disease (pneumonia), a long-lasting fever that causes the absence of the estrous cycle, painful genitalia that inhibit bulls from serving, and early-stage abortion, which happens often [26]. The strain of the *Capripoxvirus*, the breed of the host cow, and, in the case of experimental infection, the method of virus transmission and the dosage all have a role in determining the severity of clinical symptoms.

**Figure 4:**
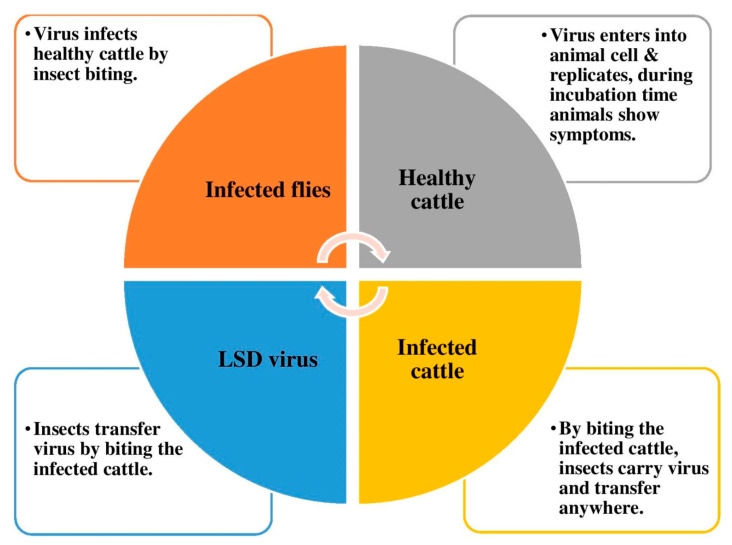
The life cycle of lumpy skin disease.

The time between anorexia and recovery is extended by lesions of the mouth, pharynx, eyes, and respiratory system. Animals that are significantly impacted experience deterioration in their general health, and under a range of circumstances, fatality rates can be substantial. For up to six months after recovery, the animals have weakness and debility. Most affected animals experience very few nodules and a smooth recovery. Even though fewer than 5% of infected animals experience chronic difficulties, LSD is a disease that has asignificant impact on production losses [27]. The complete life cycle of LSDV has been represented in Figure 4.

## 3. Diagnosis and Outbreaks of LSD

The typical clinical indications of LSD are paired with laboratory evidence of the virus or antigen’s presence to make the diagnosis [24].

Moreover, the histological traits are the most indicative of LSD, which include (a) nodules that involve all layers of the skin, subcutaneous tissue, and frequently nearby musculature and are invariably accompanied by congestion, hemorrhage, edema, vasculitis, and necrosis; (b) proliferation, edema, congestion, and hemorrhage of the lymphocytes; (c) cellular infiltrates, perivascular fibroplasia, thrombosis, and infarction; and (d) intracytoplasmic eosinophilic inclusions, which may be exhibited by various cells. Moreover, there are other methods that could be utilized for the diagnosis of LSD including (a) the isolation of virus [28], (b) electron microscopy [29], (c) agar gel immunodiffusion, (d) polymerase chain reaction (PCR) [21,22], (e) fluorescent antibody tests, (f) Enzyme-linked immunosorbent assays, and (g) real-time PCR.

### Differential Diagnosis

**Figure 5:**
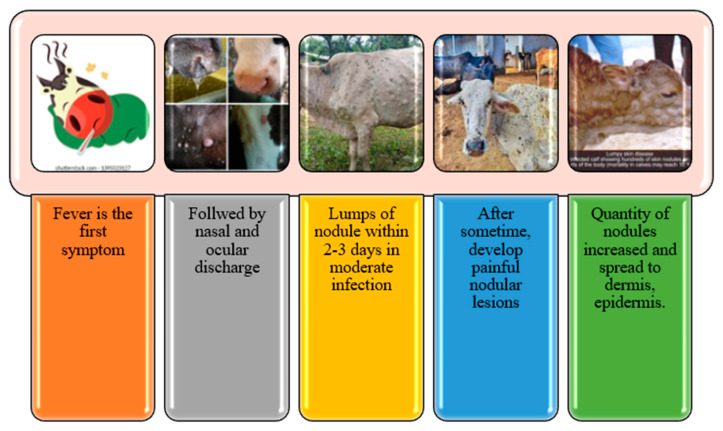
Lumpy skin disease symptoms from mild to severe.

Symptoms similar to LSD are caused by a variety of diseases (Figure 5). To provide the optimal preventative and control actions for sensitive herds, it is critical to establish a firm diagnosis. Various other conditions can be mistaken for LSD, such as pseudo-lumpy skin condition, bovine malignant catarrhal fever (Snotsiekte), demodicosis (Demodex), bovine viral diarrhea/mucosal illness, and besnoitiosis. In order to validate a provisional clinical diagnosis, reliable and quick laboratory procedures are necessary for the effective control of LSD. The main resources for such an effective epidemiological investigation are diagnostic and screening tests [29] (Table 2).

In order to identify LSD in clinically infected, fevered, and otherwise seemingly healthy dairy cows, various diagnostic techniques are used. These include viral isolation; serological tests, such as the indirect fluorescent antibody test (IFAT), the virus neutralization test (VNT), and indirect ELISA (iELISA); and molecular techniques, such as dot blot hybridization (DBH) and PCR [22]. In addition, a regular histopathological examination, immune histology staining, and agent identification can be used to create a laboratory test for LSD [35].

For both monitoring or suppressing out breaks and disease surveillance, a quick test for CaPV that can identify the virus before the development of clinical symptoms would be extremely helpful [36,37]. Real-time PCR tests offer quick and innovative ways to detect viruses in diagnostic laboratories. Real-time PCR assays have several advantages over “classical” single or nested PCR methods for diagnostic purposes, including faster and higher-throughput assays; enabling quantitative estimation in addition to “positive” or “negative” results; and, compared to conventional quantitative PCR techniques, real-time quantitative PCR is more precise and labor-efficient [36]. The considerable nucleotide sequence diversity (mismatches) in the genomes of the numerous strains of the targeted virus, however, makes the PCR-based diagnostic techniques vulnerable, despite their many benefits. The frequency of mismatches between the target and primer sequences increases, which reduces the amplification or even yields negative PCR findings [36].

A quick, accurate, and focused method for confirming *capripoxviruses*, including LSD, is real-time PCR [37]. Zeynalova et al. (2018) took two thirds of all samples from susceptible animals containing viral DNA. Skin nodule samples regularly tested positive for LSDV, while blood and organ samples were less likely to do so [38]. This aligned with a study’s findings that LSD viremia lasts for a few days; blood samples tested positive for PCR 4 to 11 days after infection, whereas skin lesions could still show signs of the virus 92 days later [35]. In a study by Kasem et al. (2018), clinical symptoms and real-time PCR were used in Saudi Arabia to identify the lumpy skin condition during the 2016 zoonosis [39]. A real-time polymerase chain reaction was used to describe the virus using tissues from skin nodules. A qPCR analysis identified the LSD virus in all tested samples (n = 191), with a very low Ct value, indicating a high concentration of the virus. A total of 64,109 cattle were investigated throughout this time, and infection, death, and case fatality rates were correspondingly 6%, 0.99%, and 16.6%. In a study conducted in Uganda, the presence of LSDV viral DNA from suspect clinical cases presenting with numerous skin nodules was confirmed using a traditional PCR with primers that targeted a 192 bp region of the LSDV P32 gene [40].

## 4. Control Measures

**Figure 6:**
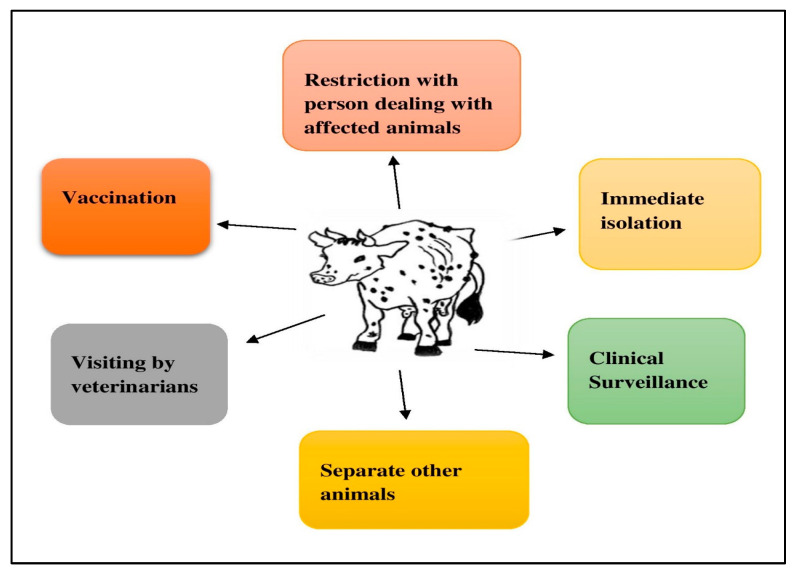
Different control measures to prevent LSD.

Because LSD is a viral illness, there is currently no specific treatment. LSD treatment is only symptomatic, with antibiotic medication used to prevent subsequent bacterial problems (Table 3 and Figure 6). Antibiotics such as penicillins, cephalosporins, tetracyclines, and fluroquinolones are prescribed for 5 to 7 days, depending on the severity of the illness. Salib and Osman started treatment studies in 2011 with the goal of reducing LSD consequences and saving lives. They were effective, utilizing a mix of medications that fight bacteria and inflammation, provide comfort, and treat infections [41,42]. They also advised to take anti-histaminic and non-steroidal anti-inflammatory medications. An anti-pyretic medication, such as paracetamol, was given to reduce fever. For anorexia recovery, multivitamins and liver-supporting medications must be taken on a regular basis [17]. However, treating LSD (and its effects) is costly and does not always result in a full recovery; as a result, avoidance is more effective for minimizing large financial losses due to hide damage; milk loss from mastitis; and losses of food products from death, miscarriage, fever, and myiasis.

### 4.1. Control of Cattle Movement

When a disease is first discovered in a country or region, the first urgent actions to be taken are a standstill and a quarantine. Zones with as few restrictions on movement as possible, as well as clinical surveillance, should be established in high-risk areas [43]. Furthermore, in order to carry out eradication treatments, including quarantine, the slaughtering of infected and in-contact animals, proper carcass disposal, cleaning and disinfecting the premises, and pest control, as soon as is practical during the eruption, prompt clinical identification is necessary [44]. However, the disease can only be controlled in endemic areas with vaccination, movement restrictions, and the euthanasia of sick animals [45]. However, cattle are sacred animals in India, so euthanasia is not allowed.

### 4.2. Vector Management

Vector control should be viewed as a supportive rather than a preventative measure because it cannot stop LSD from spreading or infecting people. The regular application of pour-on insect repellents and insecticides for cattle and buffalo, in conjunction with other pest control methods, can aid in vector control in farm buildings and grounds. The complete eradication of the disease, however, is likely to be difficult, given the participation of arthropod vectors, and any delays in removing affected animals increase the danger of LSD spread [35].

### 4.3. Vaccination

The best method for preventing the spread of LSD is the vaccination of cattle with a vaccine that has been shown to be effective, especially if given pre-emptively or before the virus enters a vulnerable region or nation. Tuppurainen et al. (2020) have started a study on the epidemiological characteristics and economic effects of lumpy skin disorders in Ethiopia, which highlights the necessity of immunization in LSD management in endemic regions [48]. Live vaccines elicit a powerful and long-lasting immune response and are effective in disease prevention [49]. However, live vaccinations can induce local inflammation and a minor illness with skin lesions [49].

*Capripoxvirus* members have been recognized to give cross-protection. Therefore, cattle can be protected against LSD infection using live-attenuated vaccinations that are homolog (Neethling LSDV strain) or hetero (sheep pox or goat pox viruses). The LSDV Neethling strain, KSGPV O-240 and O-180 strainspox (GTP) strains, and Kenyan sheep and goat pox virus commercially accessible Capripoxvirus(CaPV) vaccine strains include the Gorgan goat, Romanian SPP, and Yugoslavian RM65 sheep pox (SPP) strains [50]. According to a study on the potency of three CaPV strains against LSD in Ethiopia by Gari et al. (2010) the Gorgan GTP vaccine can successfully protect livestock against LSDV, while the Neethling and KSGP O-180 vaccines seemed to be ineffective, indicating the requirement of additional molecular diagnoses for those inefficient vaccines [51]. Earlier, there were many factors identified for vaccine failure, including the difference of strains between the vaccine and the field strain, the low titer of the vaccine, the vaccination of animals already incubating the disease, and the mishandling of the vaccine during transportation and storage [52]. Later, the lack of cross-protection with the vaccinal strain and the poor immunogenicity of the vaccine due to over-attenuation might attribute to its poor efficacy and the failure of the Neethling vaccine in Ethiopia [51,52].

Due to possible safety concerns with the use of the live-attenuated LSDV vaccine, it is advised to use the same vaccination in nations that have historically been free of LSD and that protect sheep against sheep pox [53].

Despite being costly and requiring numerous doses, inactivated vaccines are safe and can be mixed with other antigens to provide polyvalent immunizations that can be administered in areas lacking infection. Additionally, inactivated vaccines may be employed as the final phase in a scheme that initially uses live vaccines to eradicate a disease [53].

Since the LSD virus is stable and lasts a long time in the environment, long-term immunization with 100% coverage should be made mandatory for disease control and prevention. It is advisable to immunize fresh animals before bringing them to the impacted farm. At the age of 3 to 4 months, calves that have been nursed by mothers who have received vaccinations or were infected naturally should be protected. Each year, pregnant cows and breeding bulls might receive vaccinations [54].

### 4.4. Awareness

Without effective collaboration between farmers and other participants in the cattle value chain, disease control cannot be accomplished effectively. Programs to raise awareness should be directed at public and private veterinarians as well as veterinary students, farmers, herders, cattle merchants, cattle truck drivers, and artificial inseminators, both in the field and in abattoirs. Veterinary professionals and livestock workers could diagnose clinical cases quickly with the help of education, which would stop the spread of the disease [23].

## 5. Conclusions and Future Perspectives

Important animals such as cattle and buffaloes have a significant role in the global economy. Cattle and buffaloes are susceptible to the dangerous illness known as lumpy skin disease. Clinically, the illness is distinguished by characteristic nodular lesions, which mostly affect the skin and the underlying tissues of infected animals. The conjunctiva and the alimentary, respiratory, and urogenital tracts may also occasionally be involved. Before recently spreading to India and other formerly disease-free Asian nations, the illness was confined to Africa and a small number of other nations. This is concerning for the livestock rearing industry because the economics of many of these nations are built on agriculture. Lesions cause significant economic losses due to lower hide quality, chronic sickness, decreased milk output, loss of weight, abortion, infertility, and death. LSD may also result in a decline in the export of cattle and livestock-related goods. To determine the true illness prevalence, the causes of LSD’s introduction into India must be investigated, combined with epidemiological random screening in various areas. The only way to avoid the disease is vaccination, in addition to efficient quarantine measures and vector control techniques.

The disease’s recent expansion into formerly disease-free regions is evidence of its epidemiological and economic significance. Animal migrations between Middle Eastern nations should be carefully regulated by veterinary authorities due to the large borders of these nations. Additionally, the careful study of the illness’s transmission and epidemiology as well as the application of efficient preventative interventions such as immunization may lead to improved disease management. Therefore, precise and quick diagnosis in endemic areas, vaccination with the homologous strain of the LSDV, vector management, restrictions on the movement of animals, and LSDV testing of bulls used for mating are all efficient ways to inhibit further spread. When designing control plans, seasonal and climatic risk factors ought to be considered. Herdsmen, veterinarians, and livestock workers should be informed about the basics of LSDV, such as transmission, to enable them to adopt management strategies to reduce the viral attack.

## Figures and Tables

**Table 1:** Outbreaks of LSDV in different countries.

S. No.	Country/Region	Date of Outbreak	References
1.	India	2019	[10]
2.	Bhutan	2020	[11]
3.	Nepal	2020	[12]
4.	Bangladesh	2019	[13]
5.	Yemen	1995	[14]
6.	Vietnam	2020	[4]
7.	Oman	2010	[4]
8.	China	2019	[15]
9.	Zambia	1929	[2]
10.	Taiwan	2021	[16]
11.	Kuwait	1991	[4]

**Table 2:** Diagnostics used for LSD and their impacts, with pros andcons.

Sr. No.	Diagnostic Techniques Used for the Identification of LSD	Impacts on LSD	Pros	Cons	Reference
1.	Viral isolation and identification through indirect fluorescent antibody test (IFAT)	It is one of the most accurate techniques for finding LSDV in a skin sample.	It is a quantitative as well as qualitative approach.	This method’s accuracy is based on the operator’s skill.	[30]
2.	Detection using conventional PCR	It is the quickest and most reliable method for LSDV detection.	It is a substitute for the gold standard that allows for quick clinical confirmation and isolation in the absence of a live agent.	It is time-consuming, less sensitive, and involves post-PCR processing that introduces carryover contamination.	[30,31]
3.	Histopathological examination	This technique is used to characterize LSDV and acts as a confirmatory test for its identification.	It is typically a very precise diagnostic method that provides greater details about a tissue and focuses on its architecture.	Its accuracy depends on the skill of the operator.	[30,31]
4.	Immunohistochemistry test (IHC)	It is utilized to validate the presence of LSDV.	Tissue samples may be fresh or frozen, and IHC is well established and widely available. IHC provides a speedy turnaround and is reasonably priced. Because there are no live viral infections present, there is very little risk to human life.	It is extremely inexpensive, but IHC equipment is very pricey. Data quantification is especially difficult, and IHC is prone to human mistakes. It is essential that employees have the right training.	[30]
5.	Indirect enzyme-linked immunosorbent Assay (iELISA)	This method is employed to identify antibodies in cows with LSDV infections.	This method is straightforward, has a high antigen–antibody response specificity and sensitivity, is accurate because multiple examinations can be performed simultaneously without time-consuming sample preparation, and is typically safe and environmentally friendly because radioactive materials and significant amounts of organic solvents are not required. Cost-effective chemicals are used in the test.	Making antibodies is labor-intensive and expensive due to the complex technology and pricey cell culture media required to manufacture a specific antibody. Erroneous positive or negative results pose a serious concern due to insufficient blocking at the bottom of the microtiter plate immobilized with the antigen. Since an antibody is a protein, it needs to be transported and refrigerated.	[31]
6.	Transmission electron microscopy (TEM)	This method is used to evaluate skin biopsies to detect LSDV infection.	TEM can be applied in a wide range of unique research, academic, and industrial fields. Enhancements of one million times or more are seen in TEM, which provides information about element and compound structures. TEM images are crisp and detailed, and with the right training, they are easy to use. The topography, form, size, and structure of the surface can all be described in depth.	Large, expensive, labor-intensive samples that may contain artefacts during preparation must be electron-transparent, tolerant of the vacuum environment, and tiny enough to fit inside the vacuum chamber for TEM to be used. Analysis and operation also require specific abilities, appropriate housing, and management. It displays black-and-white images.	[32]
7.	Virus neutralization test (VNT)	LSD employs this technique. Neutralizing antibodies take 3–4 days to manifest after the onset of pathogenic alterations.	It is a straightforward technology with a high performance, good accuracy, and sensitivity due to an antigen–antibody interaction.	High probability of false-positive or -negative results as a result of inadequate blocking of the antigen-coated microtiter plate surfaces. Since the production of antibodies requires technical expertise and an expensive cell growth medium, they are expensive to produce.	[33]
8.	Agar gel immune diffusion test (AGID)	The procedure’s cross-reactivity with antibodies of all other poxviruses make it less precise than VNT.	It is an easy, group-specific test.	It takes time, is group-specific, has a semi-quantitative nature, and is moderately sensitive.	[33]
9.	Immunological blotting (Western blotting)	Western blotting is difficult and expensive to perform, but it is exceedingly precise.	It tests denatured proteins to find changes in their functional states; is a delicate assay; provides excellent, clear information that is easy to interpret; and there are thousands of widely available commercial antibodies.	The tissue needs to be homogenized, which takes some time.	[33]
10.	Real-time PCR (qPCR)	Real-time PCR tests offer quick and innovative ways to detect viruses in diagnostic laboratories.	Real-time PCR techniques enable quantitative estimation in addition to “positive” or “negative” results.	The considerable nucleotide sequence diversity (mismatches) in the genomes of the numerous strains of the targeted virus makes the PCR-based diagnostic techniques vulnerable, despite their many benefits.	[34]

**Table 3:** All control measures that have been used and their impacts on LSD.

Sr. No.	Different Control Measures for LSD	Impacts of Control Measures on LSD	Reference
1.	Control of animal movement	LSD prevention and reducing the economic tolls of the outbreaks.	[46]
2.	Restrictions for persons dealing with affected animals	People should not be allowed to leave the affected region if possible.	[47]
3.	Immediate isolation	The isolation of symptomatic affected animals may be carried out with all precautions and bio-security measures.	[47]
4.	Clinical surveillance	Clinical surveillance against LSD in affected areas should be intensified.	[47]
5.	Separating other animals	By separating other animals from affected animals, the spread of LSD can be controlled.	[47]
6.	Visiting by veterinarians	Regular visiting by veterinarians until all cases are recovered and taking all precautions to avoid further spread of the disease to other farms should be performed.	[47]
7.	Vaccination	The vaccination of buffalo with the available goat pox vaccine prevents LSD. Currently, we only give immunizations against the sheep and goat pox viruses. Due to the fact that all three viruses belong to the same capripoxvirus genus, these heterologous vaccinations only provide cross-protection (up to 60–70%) for cattle against LSD.	[32]

## Data Availability

This study did not report any data.
